# Quantitative CT of emphysema, wall thickness and mucus plugs in alpha-1-antitrypsin deficiency: relationship to clinical outcomes

**DOI:** 10.1007/s00330-025-12188-7

**Published:** 2025-12-09

**Authors:** Gaël Dournes, Amel Imene Hadj Bouzid, Klervi Doucet, Ilyes Benlala, Arnaud Maurac, Elodie Blanchard, Isabelle Dupin, Patrick Berger, Pauline Henrot, Maeva Zysman

**Affiliations:** 1 Univ. Bordeaux, INSERM, CRCTB, U 1045, Bordeaux, France; 2 CHU de Bordeaux, Service d’imagerie médicale, Bordeaux, France; 3 CHU de Bordeaux, Service de pneumologie, Bordeaux, France; 4 CHU de Bordeaux, Service d’Explorations Fonctionnelles Respiratoires, Bordeaux, France

**Keywords:** Chronic obstructive pulmonary disease, Artificial intelligence, CT scan, Alpha-1-antitrypsin deficiency, Exacerbation

## Abstract

**Objectives:**

Alpha-1-antitrypsin deficiency (AATD) is a rare genetic disorder leading to chronic obstructive pulmonary disease (COPD). Emphysema is the major structural damage visible on CT scans. However, there is little knowledge on the association between other structural abnormalities, such as bronchiectasis (BE), airway wall thickening (WT) or mucus plugs (MP), and clinical features.

**Materials and methods:**

Retrospective study between 2008 and 2022 at one University Hospital of Bordeaux on all consecutive AATD patients. Bronchial and parenchymal alterations were evaluated with an (artificial intelligence) AI-driven Normalized Volume of Airway Abnormalities (NOVAA-CT) scoring system, including BE, WT, MP and emphysema quantifications. We evaluated correlations between forced expiratory volume in 1-s (FEV1%), dyspnea severity through the mMRC scale and the occurrence of at least one exacerbation in the year following CT scan.

**Results:**

Fifty-two AATD patients were included (median FEV1: 47% (40–65)). CT features of BE, WT and MP were present in 100%, 94.2% and 59% of the study population, respectively, with a lower versus upper lung predominance (*p* < 0.05). WT (*p* < 0.001) and BE (*p* = 0.04) correlated with FEV1% but not mMRC (*p* ≥ 0.09). Conversely, MP did not correlate with FEV1% (*p* = 0.08) but with mMRC (*p* = 0.01). Emphysema strongly correlated with both FEV1% and mMRC (*p* < 0.001). In multivariate analysis, after adjustment for age, genotype and tobacco consumption, the best predictor of exacerbation was WT (OR = 1.12 [1.02–1.22]; *p* = 0.01).

**Conclusion:**

This study demonstrates that AI-assisted identification of structural airway abnormalities is frequent in AATD patients and carries distinct clinical significance. Among them, WT was the most robust predictor of exacerbations.

**Key Points:**

***Question*** *Emphysema is the major structural damage in alpha-1-antitrypsin deficiency (AATD). Clinical associations of bronchial abnormalities such as bronchiectasis (BE), mucus plugs (MP) and wall thickness (WT) are lacking.*

***Findings*** *Quantitative CT of BE and WT correlated with PFT (p ≤ 0.05), while MP correlated with dyspnea scale (p = 0.01). The best predictor of exacerbation was WT (OR = 1.12 [1.02–1.24]).*

***Clinical relevance*** *AI-assisted identification of bronchial abnormalities is frequent in AATD patients in addition to emphysema alone and carries distinct clinical significance. These findings highlight the importance of comprehensive CT-based evaluations to better characterize disease phenotype and guide clinical management in AATD.*

**Graphical Abstract:**

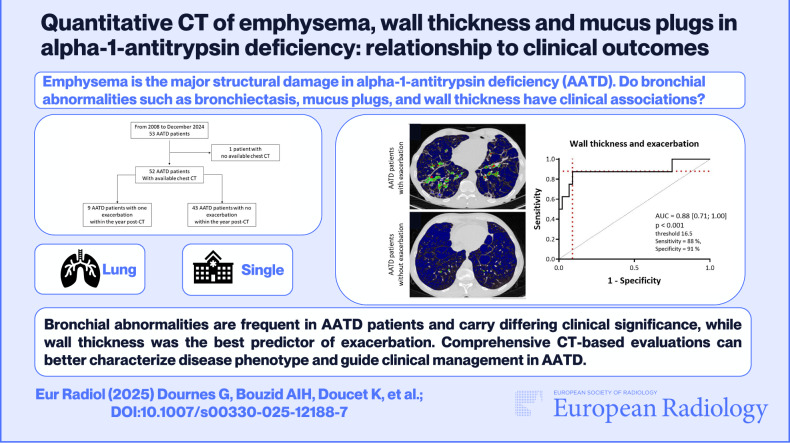

## Introduction

Alpha-1-antitrypsin deficiency (AATD) is a rare genetic disorder leading to chronic obstructive pulmonary disease (COPD). Emphysema is the major structural damage visible on Computed tomography (CT) scans and is believed to occur primarily because of an inability to inhibit neutrophil elastase released in the lung, thereby causing excessive tissue proteolysis and progressive alveolar destruction. In addition, AAT, a serine protease inhibitor, is also a potent anti-inflammatory and immunomodulatory protein, suggesting broader effects on the lungs due to immune dysregulation [1].

However, while the association between AATD and COPD/emphysema is undisputed [2, 3], the relationship between AATD and bronchial abnormalities such as bronchiectasis [4] or wall thickness is still a matter of debate [5, 6]. Indeed, the pathophysiology of AATD differs from smoking-related COPD, and it has been suggested that, consequently, the prevalence and clinical impact of such structural damages could be different [2]. Recently, there has also been interest in examining mucus plugs (MP), which have emerged as a meaningful smoking-related COPD biomarker [7, 8], such as the association between MP and accelerated lung function decline, higher exacerbation frequency, and worse overall mortality [9]. Nevertheless, previous reports indicate that the association between mucus plugs and airflow obstruction or hypoxemia in smokers is lowered, if not nullified, by the presence of emphysema [7]. Because AATD patients are prone to develop severe emphysema, the clinical impact of mucus plugs and other airway abnormalities in this setting remains unknown.

CT imaging is the gold standard in chest imaging and offers an opportunity to investigate the bronchial and parenchymal compartment of the lung in vivo. However, the bronchial tree is a complex structure, for which visual evaluation may lack reproducibility [10] while conventional quantitative measurements may be limited to a couple of bronchial paths [11]. To cope with these known shortcomings, artificial intelligence (AI)-based quantifications have been developed and were shown to reach holistic evaluation of structural alterations of airways, where all voxels of a lung CT scan can be analyzed sensitively and reproducibly [8, 12, 13].

Until now, most CT lung studies have focused on emphysema quantification, which has been used as the primary endpoint in AATD clinical trials for more than 10 years [14]. We hypothesize that, beyond emphysema alone, quantitative CT analysis of airways with AI could help clarify their association with AATD severity markers such as airflow obstruction and pulmonary exacerbation. Thus, the aims of the study were to assess (1) the prevalence of airway abnormalities in AATD according to an AI-driven detection, (2) the correlation between quantitative CT and airflow obstruction and breathlessness severity and (3) the value of AI to predict AATD exacerbations.

## Materials and methods

### Patients

We conducted a retrospective study at the University Hospital of Bordeaux between 2008 and 2024. We included all consecutive AATD patients aged 18 years or older, with at least one chest CT scan performed during follow-up (Table E1). Exclusion criteria were the absence of AATD genotyping, patients with the MZ genotype, and those who had undergone lung transplantation within the year after the CT scan. Exacerbations were defined as an acute worsening of respiratory symptoms resulting in additional therapy, oral corticosteroids and/or antibiotics. The study was conducted in accordance with French legislation and ethical codes; it complied with the protection of personal health data and the protection of privacy as specified by Article 65-2 of the amended Data Protection Act and general data protection regulations. Due to the retrospective nature of the study, informed consent was waived, and the study was approved by the local Ethics Committee (number CHUBX2024RE0125). The study design adhered to the STROBE guidelines.

The sample size was calculated by estimating the confidence interval (CI) of an area under the ROC curve (AUC). The CI was set at 95%, with an AUC of at least 0.85 with a lower bound of the 95% confidence interval of 0.70 [15, 16] to predict exacerbation from quantitative CT. According to a recent publication, 22% of AATD patients were expected to experience at least one exacerbation in the year following CT [3], and thus, the ratio between patients with and without the event was 4. Therefore, a minimum number of subjects of 40 was expected, with a minimum of 8 patients with exacerbation.

### Pulmonary function test

Plethysmography and spirometry were used to measure lung volumes and bronchial airflow limitation. The functional parameters were recorded in liters and expressed as percentages of predicted values (%pred): forced expiratory volume in 1-s (FEV1), forced vital capacity (FVC), residual volume (RV), and total lung capacity (TLC). In addition, the transfer lung capacity of carbon monoxide (TLCO) was recorded. European Respiratory Society and American Thoracic Society guidelines were chosen for reference values [17, 18].

### Quantitative CT analysis of airway structural damage

Bronchial and parenchymal alterations were evaluated fully automatically with an AI-driven Normalized Volume of Airway Abnormalities (NOVAA-CT) scoring system [12, 19–21]. Briefly, the automated outcome allows for the semantic CT segmentation of bronchiectasis, bronchial wall thickening, bronchial mucus, bronchiolar mucus, collapse/consolidation, using the standardized definition of structural abnormalities by the Fleischner Society Glossary of terms [22] and their localization (Supplementary Method E1).

Standard measurement of emphysema using the density mask principle was added [23] as an additional post-processing step, with calculation of the percentage Low Attenuation Volume (LAV%). According to reference, a threshold of 6% was used to consider LAV% significantly altered [23].

All quantitative measurements of airway and parenchymal alterations were normalized by the lung volume through CT scan as previously described [12, 20, 23], and regional evaluation of the lung lobes was allowed [24]. Thus, according to reference, NOVAA-CT is a normalized scoring system and expressed without units, while emphysema measurements are expressed as (%).

### Visual CT analysis of bronchiectasis morphology

Complementary visual evaluations were performed in addition to the quantitative automated metrics. Regarding bronchiectasis, an evaluation of bronchial dilatation (BD) patterns shown on AI labels was scored independently by two observers with 8 (IB) and 20 years of experience (G.D.) according to a previously published method [25]. Bronchiectasis morphology was considered as “cylindrical” when dilated bronchi had a relatively smooth, uniform caliber and roughly parallel walls, “cystic” when dilated bronchi had a saccular appearance (i.e., focal bronchial wall protrusion with a ballooned outline) and “varicose” when dilated bronchi had multiple indentations giving a ruffled or beaded contour.

The patterns of cylindric, cystic and varicose were scored as 0, 1 and 2, respectively, and the maximum score per patient was chosen for further analysis, allowing for discrimination of those patients with cylindrical bronchiectasis only, i.e., patients with a maximum score of 0.

### Statistical analysis

Statistical analyses were performed using R statistical software. Continuous data were presented as the median and interquartile range. The chi-squared test was used to compare categorical variables across the following subgroups: age, sex, smoking habits, lung function tests. Comparisons were analyzed by using the Mann–Whitney test and correlations by using the Spearman rank test. Missing data for symptoms, pulmonary function tests, and arterial blood gases were addressed using multiple imputation by chained equations, assuming data were missing at random. The imputation model included all clinical and functional variables considered in the analyses. Logistic multivariate analysis was performed with the occurrence of exacerbation as the dependent variable, and to account for confounding factors with an adjustment for age, genotype and tobacco consumption. We limited the number of covariates entered into the multivariate model to reduce the risk of overfitting, following the recommended rule of thumb of events per variable [26]. Only variables with strong clinical relevance or significant univariate associations were included. Then, to determine the best model to predict exacerbations, we used a forward/backward stepwise logistic regression analysis, using the occurrence of at least one exacerbation in the year following CT as the dependent binary outcome (Yes/No) [27, 28] (Supplementary Method E2). Evaluation of the area under the curve was calculated to assess the value of quantitative CT to predict exacerbation. We kept the best model only, which was found to be airway wall thickening, with an AUC of 0.88 and a lower bound of 95% CI of 0.71, which meets the a priori hypothesis and associated calculation of sample size of 0.85 with a lower bound of 0.70.

## Results

### Patients characteristics

**Fig. 1:**
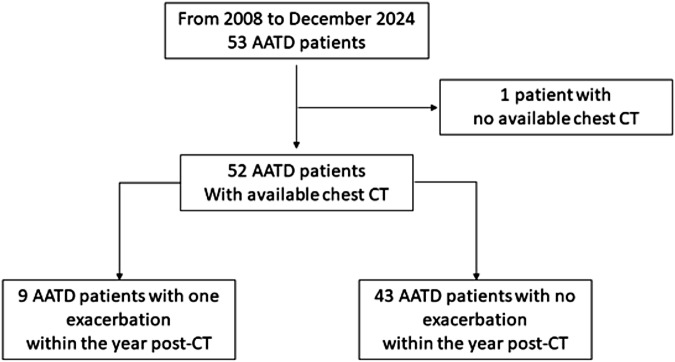
Flowchart. AATD, alpha-1-antitrypsin deficiency; CT, computed tomography

From 2008 to 2024, we included 52 AATD patients (37 ZZ genotype, 13 SZ genotype, 1 MaltonZ, 1 ZQoBolton) with available CT scan (Fig. 1). Of those, 25 were men (47%), and the overall median age was 56 [48; 63] years (Table 1). Pulmonary function tests (PFT) were median FEV1 value at 47 [40; 65] % predicted and pre-bronchodilator FEV1 1.44 [1.13; 1.77] L, TLC: 123.0 [111.8; 128.3] %, RV: 182.0 [137.8; 216.3] % and TLCO 38 [23; 53] % predicted. Seven patients presented at least one exacerbation the year before the CT scan, of whom only 3 exacerbated again the following year. Nine patients presented at least one exacerbation in the year following the CT scan; one patient had two of them (Fig. 1). All patients with exacerbator phenotype had ZZ genotype. The main demographic and clinical parameters are described in Table 1. Patients with and without exacerbation showed comparable baseline characteristics, except for the genotype (all patients with exacerbations carried ZZ genotype), and lung function parameters, which were lower in patients with exacerbations (Table 1).

**Table 1:** Patients’ characteristics at baseline

		Total population (*N* = 52)	Patients without exacerbation (*N* = 43)	Patients with ≥ 1 exacerbation (*N* = 9)
Demographic data	Age, years	56 [48; 63]	55 [48; 62]	62 [54; 68]
	Male sex	25 (47)	21 (49)	4 (44)
	BMI, kg/m^2^	23.4 [20.8; 27.1]	24.1 [21.6; 27.1]	20.0 [17.3; 24.0]*
	Current smoker	2 (4)	2 (5)	0 (0)
	Former smoker	36 (72)	30 (70)	6 (67)
	Smoking history, pack-years	13.0 [0.0; 17.5]	12.0 [0.0; 18.8]	14.0 [9.3; 15.8]
Genotype	ZZ	37 (71)	28 (65)	9 (100)*
	SZ	13 (26)	13 (30)	0*
	Rare mutations	2 (4)	2 (5)	0
Non-drug treatments	Patients on LTOT	4 (8)	2 (5)	2 (22)
	Rehabilitation within the year after CT	16 (31)	12 (28)	4 (44)
Medications	Augmentation therapy	42 (84)	35 (81)	8 (88)
	LABA	42 (84)	34 (79)	9 (100)
	ICS	33 (66)	28 (65)	6 (67)
Symptoms	≥ 1 exacerbation within the year before CT	7 (13)	5 (11)	2 (22)
	≥ 1 exacerbation within the year after CT	9 (18)	0	9 (100)*
	BODE index	2 [1; 4]^a^	2 [0; 4]^a^	2.5 [1.8; 3.3]^c^
	mMRC, points	2 [1; 3]^b^	2 [1; 3]	2 [2; 3]
Pulmonary function tests	FEV_1_, L	1.44 [1.13; 1.77]^c^	1.59 [1.25; 1.96]^c^	1.13 [0.84; 1.43]^b^
	FEV_1_, % pred	47 [40; 65]^c^	49 [42; 65]^c^	35 [27; 50]^b,*^
	FEV_1_ / FVC	45.4 [37.2; 50.1]^c^	46 [38.1; 55.2]^c^	40.6 [28.2; 50.0]^b^
	RV, %pred	182.0 [137.8; 216.3]^d^	173.5 [136.8; 208.8]^d^	209.5 [164.3; 264.0]^b^
	TLC, %pred	123.0 [111.8; 128.3]^d^	123.0 [111.0; 128.0]^d^	129.0 [120.0; 138.5]^b^
	TLCO, %pred	38 [23; 53]^e^	40.5 [23.0; 62.0]^e^	27 [18; 39]^b^
Arterial blood gases	PaO_2_, mmHg	70 [64; 77]^c^	72 [65; 77]^c^	60 [55; 67]^*^
	PaCO_2_, mmHg	36 [34; 38]^c^	36 [34; 38]^c^	38 [35; 40]
CT parameters	Normalized volumetric scoring			
	Bronchiectasis	9.5 [4.8; 20.0]	7.8 [3.2; 15.2]	47.0 [12.8; 130.2]*
	Wall thickness	5.8 [3.4; 14.4]	5.3 [2.8; 8.3]	46.7 [21.0; 156.4]*
	Mucus plug	0.1 [0.0; 0.9]	0.1 [0.0; 0.3]	4.7 [1.0; 23.8]*
	Bronchiolar mucus	0.1 [0.0; 0.2]	0.0 [0.0; 0.2]	0.9 [0.1; 4.8]
	Collapse/consolidation	1.8 [0.7; 7.4]	1.5 [0.6; 5.4]	8.8 [1.4; 20.9]*
	Emphysema quantification (LAV%)	22.5 [12; 36]	26.3 [10.6; 48.7]	53.4 [44.8; 76.2]*

Values are median [quartile 1; Quartile 3] or *n* (%) as appropriate

*BMI* body mass index, *LTOT* long-term oxygen therapy, *LABA* long-acting beta-agonist, *ICS* inhaled corticosteroids, *CT* computed tomography, *mMRC* modified Medical Research Council Dyspnea Scale, *FEV1* forced expiratory volume in 1 s, *FVC* forced vital capacity, *RV* residual volume, *TLC* total lung capacity, *TLCO* carbon monoxide diffusing capacity, *pred* predicted value, *HU* Hounsfield unit, *LAV* low attenuation volume

* *p* < 0.05

^a^ 23 missing data

^b^ 1 missing data

^c^ 4 missing data

^d^ 3 missing data

^e^ 9 missing data

### Semantic description of airway abnormality features in patients with AATD

**Fig. 2:**
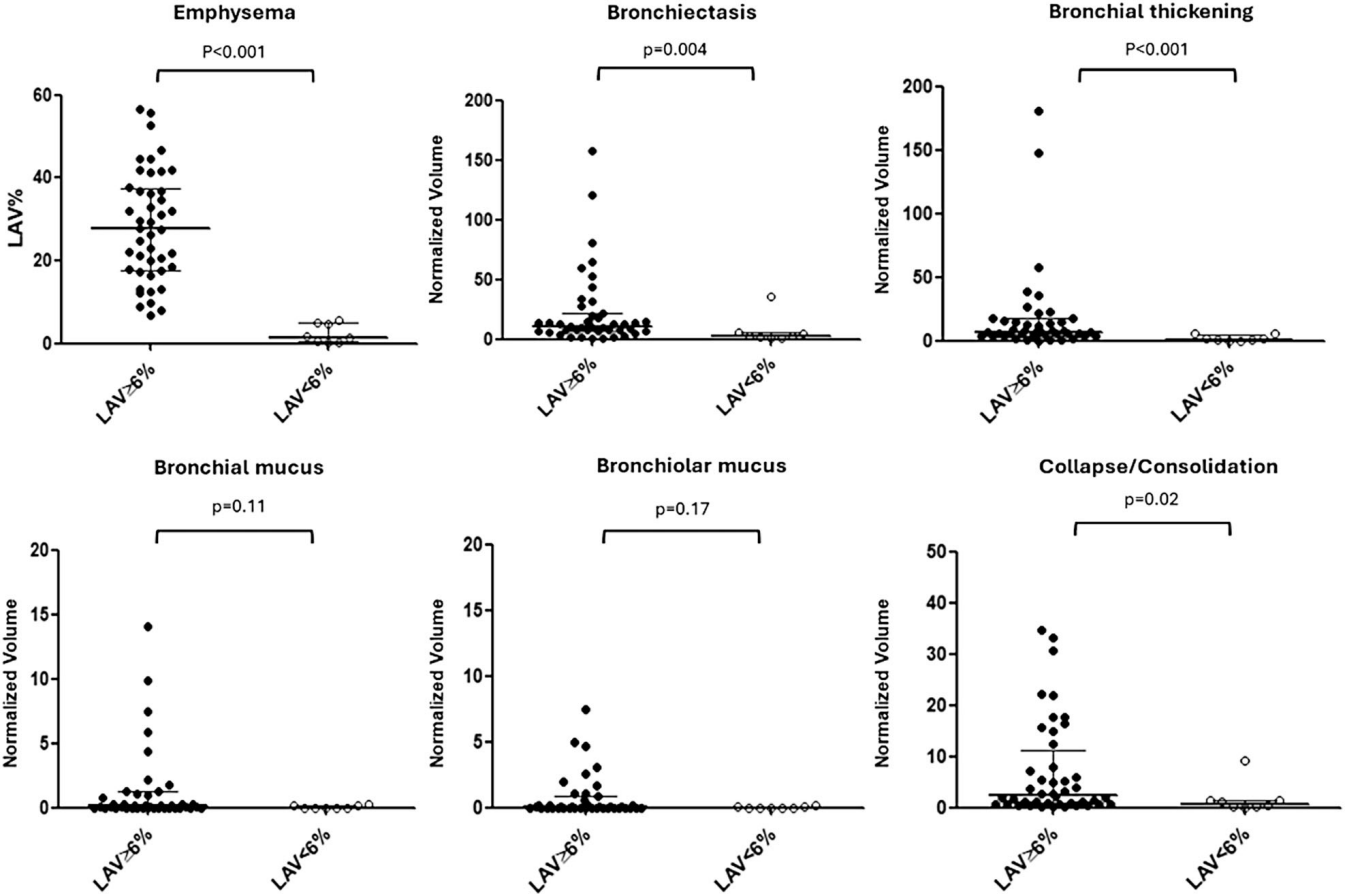
Comparison of quantitative computed tomography in AATD patients with and without emphysema. The presence of emphysema is assessed according to a threshold of 6% in percentage low attenuation volume (LAV%). Columns and bars indicate medians with interquartile range, respectively, in patients with (gray column) and without (white column) emphysema. AATD, alpha-1-antitrypsin deficiency

As expected, patients with AATD had severe emphysema, with a median LAV% of 22.5 [12; 36], ranging from 0.1 to 56.7. Eight out of 52 (15%) AATD patients had quantitative measurement of emphysema below the threshold of 6% (Table 1). These patients were also found to have lower volumes of BE (*p* = 0.004), bronchial thickening (*p* < 0.001) or collapse/consolidation (*p* = 0.02) but not that of bronchial or bronchiolar mucus (*p* ≥ 0.11) (Fig. 2).

**Fig. 3:**
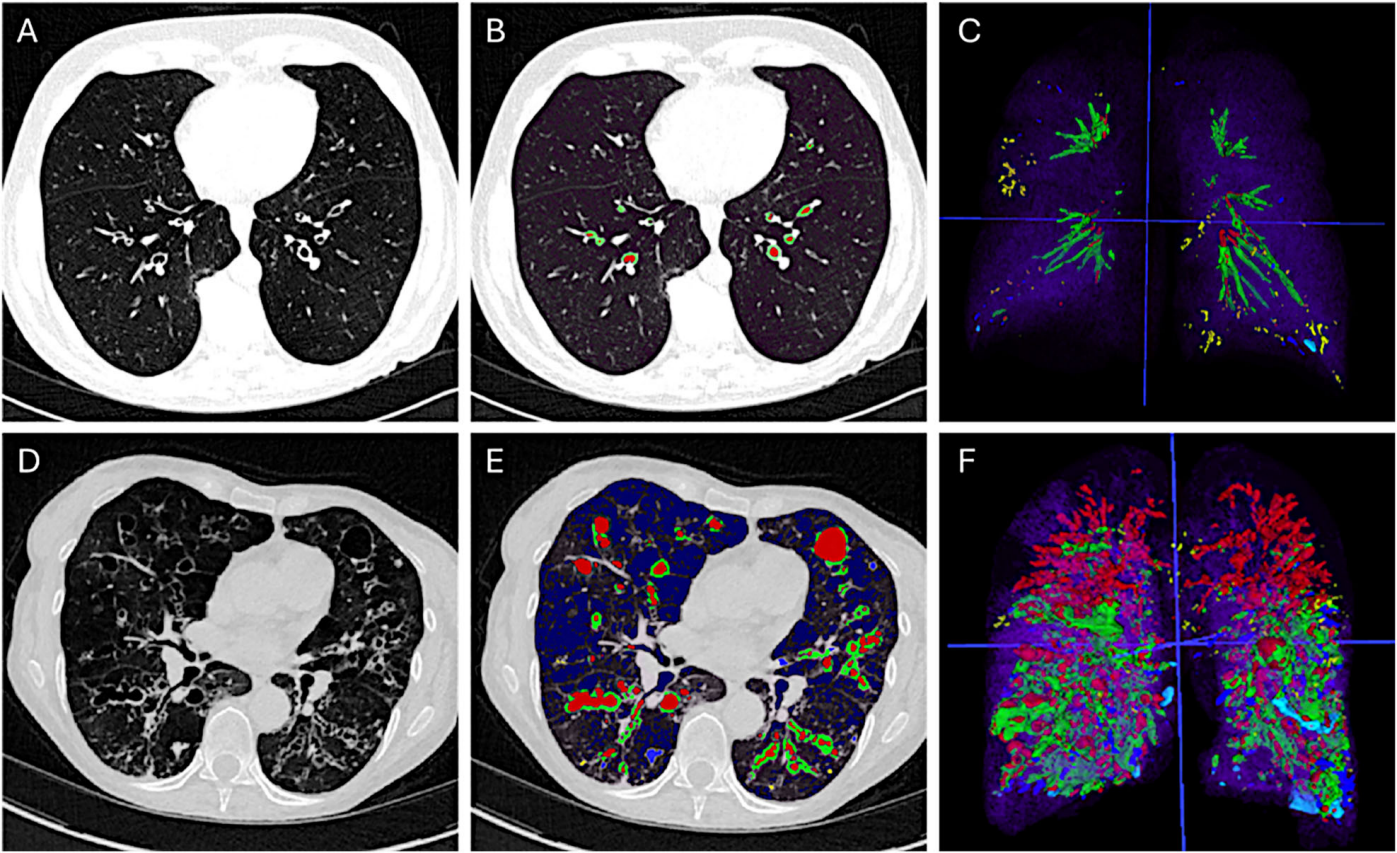
Illustration of bronchiectasis patterns found in chest CT of AATD patients. Axial CT scans (**A**, **D**) in a 68-year-old male (**A**) and 69-year-old female (**D**) with AATD are shown. Corresponding (artificial intelligence) AI-driven Normalized Volume of Airway Abnormalities (NOVAA-CT) labeling (**B**, **E**) and 3D reformation (**C**, **F**). Red, green, blue, yellow and dark blue labels correspond to the automated segmentation of bronchiectasis, wall thickness, bronchial mucus, bronchiolar mucus and emphysema, respectively. Both patients had severe emphysema, with LAV% of 27% and 28%, respectively. In the upper row, bronchiectasis shape was classified into the cylindrical pattern. In the lower row, bronchiectasis shape was classified into the cystic and varicose patterns. AATD, alpha-1-antitrypsin deficiency; LAV%, low attenuation volume

**Fig. 4:**
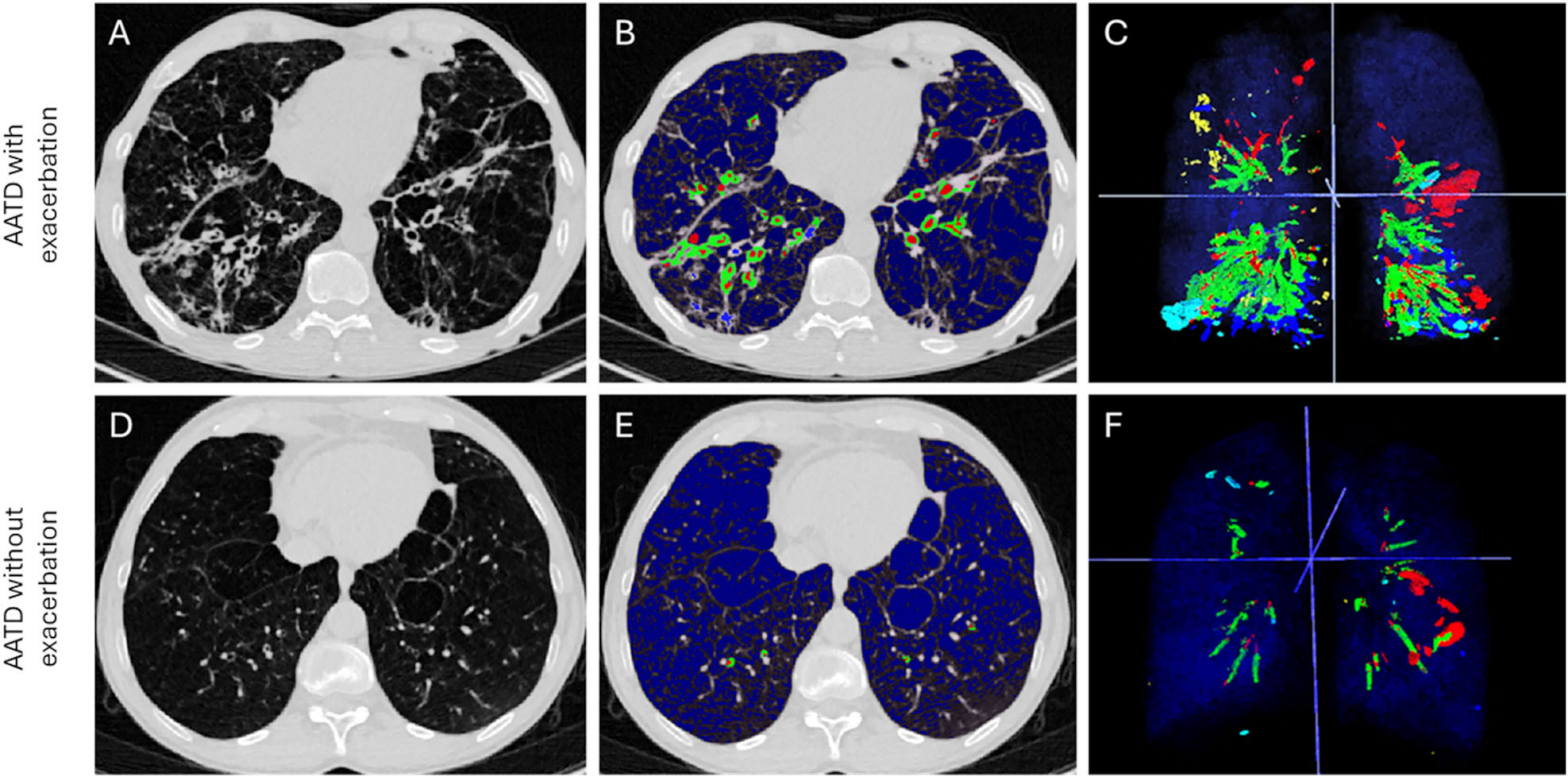
Illustration of wall thickness patterns found in chest CT scans of AATD patients with and without exacerbation in the year following CT. Axial CT scans (**A**, **D**) in a 68-year-old male (**A**) and a 73-year-old female (**D**) with AATD are shown. Corresponding (artificial intelligence) AI-driven Normalized Volume of Airway Abnormalities (NOVAA-CT) labeling (**B**, **E**) and 3D reformation (**C**, **F**). Red, green, blue, yellow and dark blue labels correspond to the automated segmentation of bronchiectasis, wall thickness, bronchial mucus, bronchiolar mucus and emphysema, respectively. In both patients with and without the occurrence of a pulmonary exacerbation in the year following CT, there is a severe amount of emphysema, assessed by LAV% of 42% and 41%, respectively. However, in the patient with subsequent exacerbation, there is marked airway wall thickening in both lung bases, with a normalized volumetric scoring of 148 versus 6 in the patients without exacerbation. AATD, alpha-1-antitrypsin deficiency; LAV%, low attenuation volume

Regarding bronchiectasis and bronchial thickening, the respective prevalence was 100% and 94.2%, indicating a high prevalence of abnormalities of central bronchi, including both lumen and wall abnormalities, in addition to emphysema, in the included patients. At visual analysis, no disagreement was found between readers to assess a maximum score of BD morphology per patient. Most patients had cylindrical BE (80.7%), followed by cystic (15.5%) and varicose (3.8%) bronchiectasis (Figs. 3, 4).

Bronchial MP were present in 31 (59%) out of 52, and bronchiolar MP in 27 (51%) out of 52. The feature of collapse/consolidation was also present in 51 out of 52 (98%) of the population study. At regional analysis, all instances of emphysema lesions and bronchial abnormalities had a lower lobes predominance as compared to the upper and middle lobes (*p* ≤ 0.01) (Supplementary Table E2).

### Correlations between CT scan features and pulmonary function test and dyspnea severity

Emphysema, as defined by a quantitative threshold of 6% of the LAV% value, had strong correlations with both PFT parameters (*p* ≤ 0.001) and mMRC (*p* = 0.01) as expected (Table 2). Concerning airway abnormalities, the normalized volumes of bronchiectasis and bronchial thickening significantly and positively correlated with PFT parameters such as FEV1%, FEV1/FVC (*p* ≤ 0.01), TLC, RV and TLCO% (Table 2). However, they did not significantly correlate with mMRC (*p* ≥ 0.09).

**Table 2:** Correlations between quantitative measurements, pulmonary functional tests and dyspnea severity score

		FEV1%	FEV1/FVC	RV%	TLC%	TLCO%	mMRC
Bronchiectasis	ρ	**−0.28**	**−0.35**	**0.29**	**0.34**	**−0.54**	0.16
	*p*-value	**0.04**	**0.01**	**0.04**	**0.01**	**< 0.001**	0.46
Wall thickness	ρ	**−0.52**	**−0.44**	**0.47**	**+0.32**	**−0.41**	0.24
	*p*-value	**< 0.001**	**0.001**	**< 0.001**	**0.02**	**0.01**	0.09
Bronchial mucus	ρ	−0.25	−0.11	0.16	0.14	−0.20	**0.34**
	*p*-value	0.08	0.45	0.26	0.34	0.19	**0.01**
Bronchiolar mucus	ρ	−0.26	−0.07	0.16	−0.02	0.05	**0.29**
	*p*-value	0.07	0.65	0.27	0.89	0.76	**0.04**
Collapse/consolidation	ρ	−0.22	−0.15	0.27	0.18	−0.30	0.12
	*p*-value	0.12	0.30	0.058	0.21	0.052	0.39
Emphysema (< 950 UH)	ρ	**−0.57**	**−0.75**	**0.53**	**0.66**	**−0.74**	**0.38**
	*p*-value	**< 0.001**	**< 0.001**	**< 0.001**	**< 0.001**	**< 0.001**	**0.01**

Data are rho correlations of Spearman. A *p*-value < 0.05 was considered significant

*FEV1* forced expiratory volume in 1 s, *FVC* forced vital capacity, *mMRC* modified Medical Research Council, *RV* residual volume, *TLC* total lung capacity, *TLCO* transfer lung capacity of carbon monoxide, *HU* Hounsfield unit

Bold font indicates significant values

Conversely, the features of bronchial and bronchiolar mucus were not significantly correlated with PFT parameters (*p* ≥ 0.08), although a significant correlation with mMRC was found (*p* = 0.01) (Table 2), confirming the positive association between mucus plugging and dyspnea and the absence of correlation with lung function. No significant correlation with PFT parameters or mMRC was noticed with collapse/consolidation.

### Prediction of exacerbation in patients with AADT

After automatic analyses with NOVAA-CT scoring system, bronchial and parenchymal alterations were significantly different between patients with (Fig. 3) and those without exacerbation in the year following the CT scan (Fig. 4). Variables thought to predict the occurrence of at least one exacerbation in the year following CT scan were evaluated, including age, sex, FEV1%, visual CT bronchiectasis morphology, visual CT mucus score, and quantitative CT measurements of bronchial abnormalities and emphysema (Table 3).

**Table 3:** Univariate and multivariate analysis of factors associated with the presence or absence of pulmonary exacerbation in the year following the CT scan

	Univariate analysis			Multivariate analysis		
With/without exacerbation	OR	95% CI	*p*-value	OR	95% CI	*p*-value
Age	1.04	[0.95; 1.13]	0.33	-	-	-
Sex	1.54	[0.30; 7.7]	0.59	-	-	-
FEV1%	0.95	[0.89; 1.01]	0.10	-	-	-
mMRC	1.26	[0.59; 2.69]	0.53	-	-	-
Visual CT analysis						
Bronchiectasis pattern	**9.1**	**[2.1; 38]**	**0.002**	-	-	-
Quantitative CT analysis						
Bronchiectasis	**1.03**	**[1.00; 1.07]**	**0.01**	-	-	-
Wall thickness	**1.12**	**[1.02; 1.22]**	**0.01**	**1.12**	**[1.02; 1.22]**	**0.01**
Bronchial mucus	**1.32**	**[1.02; 1.71]**	**0.03**	-	-	-
Bronchiolar mucus	1.01	[0.92; 1.1]	0.75	-	-	-
Collapse/consolidation	1.07	[0.99; 1.15]	0.057	-	-	-
Emphysema	**1.07**	**[1.01; 1.14]**	**0.02**	-	-	-

Data are odds ratios with 95% confidence interval. The bronchiectasis pattern corresponds to cylindrical versus any other patterns, such as cystic or varicose

*CI* confidence interval, *CT* computed tomography, *FEV1* forced expiratory volume in 1 s, *mMRC* modified Medical Research Council, *OR* odds ratio

Bold value, for significant values

In univariate analysis, visual bronchiectasis morphology (OR = 9.1 [2.1; 38]; *p* = 0.002), bronchiectasis (OR = 1.03 [1.00; 1.07]; *p* = 0.03), bronchial thickening (OR = 1.12 [1.02; 1.22]; *p* = 0.01), bronchial mucus (OR = 1.32 [1.02; 1.71]; *p* = 0.01) and emphysema presence (OR = 1.07 [1.00; 1.14]; *p* = 0.02) were independent predictors of acute exacerbation (Table 3).

In multivariate stepwise regression analysis, considering multicollinearity, only bronchial thickening remained independently associated with the presence or absence of exacerbation (OR = 1.12 [1.02–1.22]; *p* = 0.01; Nagelkerke R^2^ = 0.55, *p* < 0.001; Table 3). The association remained significant when adjusting for other covariates thought to relate to AATD exacerbation, such as age, genotype, or tobacco consumption or medication use.

**Fig. 5:**
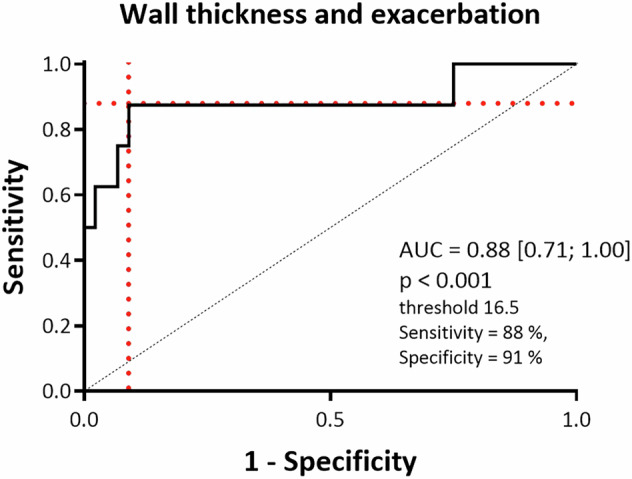
A receiver operating characteristic (ROC) curve was generated to evaluate the ability of wall thickness to discriminate between patients with and without exacerbation. The area under the curve (AUC) was calculated to assess the predictive performance

A receiver operating characteristic (ROC) curve was constructed to assess the discriminative power of airway wall thickening in identifying patients with a history of exacerbations. The analysis yielded an area under the curve (AUC) of 0.88 (95% CI: 0.71–1.00), indicating excellent predictive performance. The optimal threshold for WT was determined to be 16.5, corresponding to a sensitivity of 88% and a specificity of 91% for detecting exacerbator patients (Fig. 5).

## Discussion

In this study involving 52 patients with AATD, we identified a high prevalence of airway and parenchymal abnormalities, underscoring the phenotypic complexity of the disease. Bronchiectasis was present in all patients, while airway wall thickness and mucus plugging were observed in 94.2% and 59% of the patients, respectively, with a predominant lower lobe distribution. Beyond emphysema, bronchial wall and lumen modifications are related to clinically relevant outcomes such as airflow limitation and dyspnea severity. Moreover, CT structural abnormalities were found to be strong predictors of pulmonary exacerbation, whereas other markers of lung severity, such as PFT and dyspnea scale, did not.

AATD is a rare genetic form of COPD. The occurrence and clinical significance of bronchial abnormalities in patients with AATD is currently not well-known, and it is unclear to what extent such bronchial abnormalities may have a clinical impact, in addition to the well-established emphysema burden. There are also recent reports that mucus plugs may play a role in COPD severity, although such evaluation has not been reported in AATD yet. To decipher the complexity of bronchial heterogeneity with sensitivity and reproducibility, we used an AI approach on CT scans, enabling a holistic evaluation of all bronchial paths and parenchymal structural alterations quantitatively.

First, emphysema, as expected, showed strong correlations with both PFT parameters and mMRC, reinforcing its central role in the pathophysiology of AATD-related lung disease. However, emphysema was not significantly associated with exacerbations in multivariate analysis, despite its strong univariate associations with functional impairment. This finding may reflect the multifactorial nature of exacerbation, where airway involvement, particularly wall thickness, plays a more significant role than parenchymal destruction alone.

Regarding bronchiectasis, we found a 100% prevalence in this population study. Interestingly, bronchiectasis was noticed even in the subset of patients without emphysema, as defined by a quantitative threshold of 6% of the LAV% value [23]. Also, bronchiectasis, together with wall thickness, was more pronounced in patients with emphysema than in those without and shared a similar bi-basal distribution. In literature, reported prevalence varies widely, ranging from 36% [29] to 96% [5]. A systematic screening among patients with bronchiectasis has shown that the frequency of AATD was not different from the general UK [30, 31] or French [32] populations. In contrast, bronchiectasis has been reported frequently in patients with known AATD [29, 33]. Several definitions of bronchiectasis are possible. In this evaluation, we used the inner lumen as reference and a threshold of a broncho-arterial ratio ≥ 1, which is a standard criterion that has been widely used in the literature, while other studies used a ratio of 1.5 [7, 34]. Moreover, the AI-driven method was able to assess all visible bronchial paths, unlike visual scorings that summarize the CT information into lobar scores. Nevertheless, from a clinical perspective, we did find that, using these methodological choices, the quantitative assessment of bronchiectasis had clinical significance regarding the relationship to airflow obstruction. On visual analysis, most patients had mild cylindrical bronchiectasis. In contrast, cystic or varicose bronchiectasis patterns were associated with increased odds of experiencing an exacerbation within the year following the CT scan.

Regarding mucus plugging, we found that it was not significantly associated with FEV₁% but correlated with dyspnea severity (mMRC), suggesting that mucus retention may contribute more to symptom burden than to spirometry alteration. Although a previous report on smoking-related COPD reported a significant relationship between FEV1% or hypoxemia and a visual mucus score [9], it is noteworthy that such a relationship was modified by the presence of emphysema. Indeed, a lower association was noticed at higher emphysema levels. Accordingly, the majority of our AATD population had a severe median emphysema extent of 22%, which accounted for less than 10% of the smoking-related COPD population described by Dunican et al, a cohort that included only a few GOLD IV patients and also patients without COPD [7]. Therefore, our findings appear to confirm that emphysema may counteract the relationship between FEV1% and mucus plugs, since the main determinant of this global measurement is more likely to be linked to diffuse functional impairment than a localized airway occlusion. However, in agreement with previous reports, we found an association with mucus plugs to predict pulmonary exacerbation [35] at univariate analysis, supporting the rationale to explore this feature even in patients with AATD.

Regarding airway wall thickness, one of our main findings relates to the pivotal role of wall thickness in AATD. Moreover, wall thickness emerged as the strongest predictor of acute exacerbations in our cohort. This finding is consistent with pathological evidence showing that airway thickness, including smooth muscle hypertrophy, mucous gland hyperplasia, and peribronchial inflammation, predisposes to increased airflow obstruction and heightened susceptibility to infectious or inflammatory triggers in smoking-related COPD [36]. In AATD, there is early and persistent neutrophil-mediated airway inflammation, mucus gland hyperplasia and structural remodeling even before overt functional decline (as shown in AATD subjects with ‘normal’ lung function [37]). Moreover, the frequent presence of airway disease and bronchiectasis (EARCO registry) in PiZZ patients suggests that thickened airway walls may promote microbial retention, impaired clearance and heightened inflammatory responses, thus predisposing to exacerbations [29]. This major component of structural bronchial alteration showed significant correlations with all PFT results, including FEV1%, FEV1/FVC, RV%, TLC% and TLCO%, highlighting its association with airflow limitation and air trapping. In agreement with previous reports, Yamashiro et al [11] and Parr et al [5] also found a relationship between airway wall thickness, measured in 2D, and FEV1%. However, the relationship with other clinical outcomes was not reported. In this study, we found that, at multivariate analysis, the best model fitting the occurrence of pulmonary exacerbation was based on the airway wall volume, even after adjusting for age, genotype, and tobacco exposure. Conversely, functional evaluations of FEV1% and mMRC did not. Also, emphysema was a predictor at univariate analysis but not at multivariate analysis. In any case, this finding suggests that wall thickness may play a more central role than emphysema in determining clinical outcomes such as exacerbations in AATD. Indeed, in AATD patients with underlying severe emphysema, the result indicates that the additional presence of an increased airway wall thickening is more closely related to the risk of developing an exacerbation in the year following CT. This finding may call for a personalized strategy to prevent this major pejorative outcome.

The study had several limitations. Given the single-center retrospective design and the limited number of acute exacerbation events, selection bias cannot be excluded, and the generalizability of our findings remains restricted. In addition, although the statistics allow us to demonstrate a significant relationship between airway wall thickness and exacerbation, the study was not designed or sampled enough to address whether a combination of other variables could further enhance the power of the prediction in the form of a multivariate model. Therefore, the findings should be interpreted with caution, given the limited number of acute exacerbation events, and larger multicenter studies would strengthen the generalization of the results. In addition, although multiple imputation was applied to account for missing data, residual bias cannot be excluded. Nevertheless, sensitivity analyses using complete case datasets yielded consistent results, supporting the robustness of our findings. Exacerbations were followed up over 1 year only, and their cumulative occurrence over the long term may be investigated. Biomarkers of lung or bronchial inflammation in blood or sputum samples were not collected to further explore the relations between the CT findings and inflammatory or microbiological modifications.

## Conclusion

Central airway structural damage is often present in patients with AATD in addition to emphysema. Such structural airway abnormalities had distinct associations with either FEV1%p or dyspnea severity. Moreover, in these patients with background emphysema, the additional presence of wall thickness was found to be the best predictor of pulmonary exacerbation.

## Supplementary information

Supplementary information

## Abbreviations

%p: Percentage predicted
AATD: Alpha-1-antitrypsin deficiency
AI: Artificial intelligence
AUC: Area under the curve
BE: Bronchiectasis
COPD: Chronic obstructive pulmonary disease
CT: Computed tomography
mMRC: Modified Medical Research Council
MP: Mucus plugging
NOVAA-CT: Normalized volume of airway abnormalities at CT
PFT: Pulmonary function test
ROC: Receiver operating characteristic
RV: Residual volume
TLC: Total lung capacity
TLCO: Transfer lung capacity of carbon monoxide
WT: Bronchial wall thickening

## References

[1] Bergin DA, Reeves EP, Meleady P et al (2010) α-1 Antitrypsin regulates human neutrophil chemotaxis induced by soluble immune complexes and IL-8. J Clin Invest 120:4236–4250. 10.1172/JCI41196 21060150 10.1172/JCI41196 PMC2993580

[2] Smith DJ, Ellis PR, Turner AM (2021) Exacerbations of lung disease in alpha-1 antitrypsin deficiency. Chronic Obstr Pulm Dis 8:162–176. 10.15326/jcopdf.2020.0173 33238089 10.15326/jcopdf.2020.0173 PMC8047608

[3] Faria N, Gomes J, Guimarães C, Marçôa R, Ferraz B, Sucena M (2024) Predicting exacerbations in alpha-1 antitrypsin deficiency using clinical and pulmonary function tests: Portuguese EARCO Registry. Respiration 103:317–325. 10.1159/000537759 38531325 10.1159/000537759

[4] Polverino E, Dimakou K, Hurst J et al (2018) The overlap between bronchiectasis and chronic airway diseases: state of the art and future directions. Eur Respir J 52:1800328. 10.1183/13993003.00328-2018 30049739 10.1183/13993003.00328-2018

[5] Parr DG, Guest PG, Reynolds JH, Dowson LJ, Stockley RA (2007) Prevalence and impact of bronchiectasis in alpha1-antitrypsin deficiency. Am J Respir Crit Care Med 176:1215–1221. 10.1164/rccm.200703-489OC 17872489 10.1164/rccm.200703-489OC

[6] Sanduzzi A, Ciasullo E, Capitelli L, Zamparelli SS, Bocchino M (2020) Alpha-1-antitrypsin deficiency and bronchiectasis: a concomitance or a real association? Int J Environ Res Public Health 17:2294. 10.3390/ijerph17072294 32235324 10.3390/ijerph17072294 PMC7178111

[7] Dunican EM, Elicker BM, Henry T et al (2021) Mucus plugs and emphysema in the pathophysiology of airflow obstruction and hypoxemia in smokers. Am J Respir Crit Care Med 203:957–968. 10.1164/rccm.202006-2248OC 33180550 10.1164/rccm.202006-2248OC PMC8048745

[8] van der Veer T, Andrinopoulou E-R, Braunstahl G-J et al (2025) Association between automatic AI-based quantification of airway-occlusive mucus plugs and all-cause mortality in patients with COPD. Thorax 80:105–108. 10.1136/thorax-2024-221928 39638548 10.1136/thorax-2024-221928 PMC12052038

[9] Diaz AA, Orejas JL, Grumley S et al (2023) Airway-occluding mucus plugs and mortality in patients with chronic obstructive pulmonary disease. JAMA 329:1832–1839. 10.1001/jama.2023.2065 37210745 10.1001/jama.2023.2065 PMC10201404

[10] Calder AD, Bush A, Brody AS, Owens CM (2014) Scoring of chest CT in children with cystic fibrosis: state of the art. Pediatr Radiol 44:1496–1506 25164326 10.1007/s00247-013-2867-y

[11] Yamashiro T, Matsuoka S, Estépar RSJ et al (2009) Quantitative airway assessment on computed tomography in patients with alpha1-antitrypsin deficiency. COPD 6:468–477. 10.3109/15412550903341521 19938971 10.3109/15412550903341521 PMC2945281

[12] Dournes G, Hall CS, Willmering MM et al (2022) Artificial intelligence in computed tomography for quantifying lung changes in the era of CFTR modulators. Eur Respir J 59:2100844. 10.1183/13993003.00844-2021 34266943 10.1183/13993003.00844-2021

[13] Dournes G, Zysman M, Benlala I, Berger P (2024) [CT imaging of chronic obstructive pulmonary disease: what aspects and what role?]. Rev Mal Respir 41:738–750. 10.1016/j.rmr.2024.10.002 39488460 10.1016/j.rmr.2024.10.002

[14] Chapman KR, Burdon JGW, Piitulainen E et al (2015) Intravenous augmentation treatment and lung density in severe α1 antitrypsin deficiency (RAPID): a randomised, double-blind, placebo-controlled trial. Lancet 386:360–368. 10.1016/S0140-6736(15)60860-1 26026936 10.1016/S0140-6736(15)60860-1

[15] White N, Parsons R, Collins G, Barnett A (2023) Evidence of questionable research practices in clinical prediction models. BMC Med 21:339. 10.1186/s12916-023-03048-6 37667344 10.1186/s12916-023-03048-6 PMC10478406

[16] Hanley JA, McNeil BJ (1982) The meaning and use of the area under a receiver operating characteristic (ROC) curve. Radiology 143:29–36. 10.1148/radiology.143.1.7063747 7063747 10.1148/radiology.143.1.7063747

[17] Miller MR, Hankinson J, Brusasco V (2005) Standardisation of spirometry. Eur Respir J 26:319–338 16055882 10.1183/09031936.05.00034805

[18] Wanger J, Clausen JL, Coates A et al (2005) Standardisation of the measurement of lung volumes. Eur Respir J 26:511–522. 10.1183/09031936.05.00035005 16135736 10.1183/09031936.05.00035005

[19] Hadj Bouzid AI, Bui S, Benlala I et al (2025) Artificial intelligence-driven volumetric CT outcome score in cystic fibrosis: longitudinal and multicenter validation with/without modulators treatment. Eur Radiol 35:815–827. 10.1007/s00330-024-11019-5 39150489 10.1007/s00330-024-11019-5

[20] Bouzid AIH, De Senneville BD, Baldacci F et al (2024) CT evaluation of 2D and 3D holistic deep learning methods for the volumetric segmentation of airway lesions. In: Proceedings of the 2024 IEEE international symposium on biomedical imaging (ISBI). IEEE, Athens, pp 1–5. Available via https://ieeexplore.ieee.org/document/10635201. Accessed 18 May 2025

[21] Bouzid AIH, Baldacci F, De Senneville BD et al (2025) 3D semantic segmentation of airway abnormalities on UTE-MRI with reinforcement learning on deep supervision. In: Proceedings of the 2025 IEEE 22nd international symposium on biomedical imaging (ISBI). IEEE, Houston, pp 1–5. Available via https://ieeexplore.ieee.org/document/10981041. Accessed 18 May 2025

[22] Bankier AA, MacMahon H, Colby T et al (2024) Fleischner Society: glossary of terms for thoracic imaging. Radiology 310:e232558. 10.1148/radiol.232558 38411514 10.1148/radiol.232558 PMC10902601

[23] San José Estépar R, Barr RG, Fain SB et al (2025) The use of CT densitometry for the assessment of emphysema in clinical trials: a position paper from the Fleischner Society. Am J Respir Crit Care Med 211:709–728. 10.1164/rccm.202410-2012SO 40126404 10.1164/rccm.202410-2012SO PMC12091028

[24] Park J, Yun J, Kim N et al (2020) Fully automated lung lobe segmentation in volumetric chest CT with 3D U-Net: validation with intra- and extra-datasets. J Digit Imaging 33:221–230. 10.1007/s10278-019-00223-1 31152273 10.1007/s10278-019-00223-1 PMC7064651

[25] Mills DR, Masters IB, Yerkovich ST et al (2024) Radiographic outcomes in pediatric bronchiectasis and factors associated with reversibility. Am J Respir Crit Care Med 210:97–107. 10.1164/rccm.202402-0411OC 38631023 10.1164/rccm.202402-0411OC

[26] Vittinghoff E, McCulloch CE (2007) Relaxing the rule of ten events per variable in logistic and Cox regression. Am J Epidemiol 165:710–718. 10.1093/aje/kwk052 17182981 10.1093/aje/kwk052

[27] Peduzzi P, Concato J, Kemper E, Holford TR, Feinstein AR (1996) A simulation study of the number of events per variable in logistic regression analysis. J Clin Epidemiol 49:1373–1379. 10.1016/s0895-4356(96)00236-3 8970487 10.1016/s0895-4356(96)00236-3

[28] Grewal R, Cote JA, Baumgartner H (2004) Multicollinearity and measurement error in structural equation models: implications for theory testing. Mark Sci 23:519–529. 10.1287/mksc.1040.0070

[29] Stockley RA, Pye A, De Soyza J et al (2023) The prevalence of bronchiectasis in patients with alpha-1 antitrypsin deficiency: initial report of EARCO. Orphanet J Rare Dis 18:243. 10.1186/s13023-023-02830-2 37573351 10.1186/s13023-023-02830-2 PMC10422747

[30] Pasteur MC, Helliwell SM, Houghton SJ et al (2000) An investigation into causative factors in patients with bronchiectasis. Am J Respir Crit Care Med 162:1277–1284. 10.1164/ajrccm.162.4.9906120 11029331 10.1164/ajrccm.162.4.9906120

[31] Carreto L, Morrison M, Donovan J et al (2020) Utility of routine screening for alpha-1 antitrypsin deficiency in patients with bronchiectasis. Thorax 75:592–593. 10.1136/thoraxjnl-2019-214195 32303623 10.1136/thoraxjnl-2019-214195 PMC7361016

[32] Cuvelier A, Muir JF, Hellot MF et al (2000) Distribution of alpha(1)-antitrypsin alleles in patients with bronchiectasis. Chest 117:415–419. 10.1378/chest.117.2.415 10669684 10.1378/chest.117.2.415

[33] Parr DG, Dirksen A, Piitulainen E, Deng C, Wencker M, Stockley RA (2009) Exploring the optimum approach to the use of CT densitometry in a randomised placebo-controlled study of augmentation therapy in alpha 1-antitrypsin deficiency. Respir Res 10:75. 10.1186/1465-9921-10-75 19678952 10.1186/1465-9921-10-75 PMC2740846

[34] Aliberti S, Goeminne PC, O’Donnell AE et al (2022) Criteria and definitions for the radiological and clinical diagnosis of bronchiectasis in adults for use in clinical trials: international consensus recommendations. Lancet Respir Med 10:298–306. 10.1016/S2213-2600(21)00277-0 34570994 10.1016/S2213-2600(21)00277-0

[35] Li X, Feng S, Yang Y et al (2025) Association between airway mucus plugs and risk of moderate-to-severe exacerbations in patients with COPD: results from a Chinese prospective cohort study. Chest. 10.1016/j.chest.2025.03.026 10.1016/j.chest.2025.03.026 40210091

[36] Hogg JC, Chu F, Utokaparch S et al (2004) The nature of small-airway obstruction in chronic obstructive pulmonary disease. N Engl J Med 350:2645–2653. 10.1056/NEJMoa032158 15215480 10.1056/NEJMoa032158

[37] Kokturk N, Khodayari N, Lascano J, Riley EL, Brantly ML (2023) Lung inflammation in alpha-1-antitrypsin deficient individuals with normal lung function. Respir Res 24:40. 10.1186/s12931-023-02343-3 36732772 10.1186/s12931-023-02343-3 PMC9893669

