# Supplementary material for: Quantitative CT of emphysema, wall thickness and mucus plugs in alpha-1-antitrypsin deficiency: relationship to clinical outcomes
Source: Eur Radiol. 2025 Dec 9;36(5):4098–109. doi: 10.1007/s00330-025-12188-7 (PMC13086677; doi:10.1007/s00330-025-12188-7)
Supplement: Supplementary file 1 — Supplementary information [file 330_2025_12188_MOESM1_ESM.pdf]

Quantitative CT of emphysema, wall thickness and mucus plugs in alpha-1-antitrypsin deficiency: relationship to clinical outcomes

ELECTRONIC SUPPLEMENTARY MATERIAL

Method E1. Description of the AI-driven NOVAA-CT scoring system framework.

Normalized Volume of Airway Abnormalities at CT (NOVAA-CT) scoring system was done according to a previously validated method for labeling five main hallmarks of chronic airway disease [1–3], i.e. bronchiectasis, peribronchial thickening, bronchial mucus, bronchiolar mucus, and collapse/consolidation.

In the NOVAA-CT holistic implementation, bronchiectasis refers to the mucus-free airway lumen dilatation, and the bronchial mucus plug was scored when a secretion filled the bronchial lumen entirely. A sixth label was also created, which corresponds to the lung parenchyma, as the total lung minus the sum of other abnormal labels.

The Total Abnormal Volume was defined as the sum of the five structural alteration volumes per CT scan.

The Total Lung Volume was defined as the sum (Total Abnormal Volume + Lung Parenchyma Volume).

To consider variations in lung volumes, notably between children and adults, normalization was performed as follows:

Normalized Volume of Label(y) = [Volume of Label(y) / Total Lung Volume] x 10^4.

The factor 10^4 was done to take into account the expected magnitude of volume difference between the normal central airway tree at the segmental level and the lung volume [4].

The 3D nnUnet architecture has been previously described in [1, 5].

The 3D nnU-Net was implemented on a system operating on Ubuntu 18.04. The environment was set up with Python 3.9, using PyTorch 2.0 and CUDA 11. The system's hardware was anchored by an Intel Xeon Gold 5217 CPU, featuring 2 physical processors and a total of 32 threads, 30 of which were allocated exclusively to nnU-Net during training. The system was bolstered by 200GB of RAM and employed a Quadro RTX 8000 GPU with 48GB of VRAM.

As previously described, several methodological features were implemented to improve the generalizability of the model. First, the AI model was trained with a total of 250 000 Training samples (including data augmentation) which is a large dataset over a wide range to CT scan manufacturers and brands, CT dose and techniques [1, 5].

Second, out of the data augmentation techniques, quantitative measurements are done on CT examinations reconstructed with standards kernels. Standard kernels are suitable for quantitative imaging of airways [6–8]. In addition, they are robust against noise and are comparable across CT machine brands, to enhance the generalizability of the quantitative technique further, across CT vendors [9, 10].

Method E2. Stepwise Regression Analysis

To determine the best model to predict chronic bronchiectasis reversibility, we used a forward/backward stepwise logistic regression analysis, using BE reversibility as the dependent binary outcome (Yes/No)[11]. The method was not testing the multivariate models by including all variables altogether. Conversely, we carefully checked for multicollinearity by using a forward/backward method, where variables are tested, entered or exit from the model in case of multicollinearity. Therefore, the remaining variables are free from possible collinearity that would bias the final model, thanks to the stepwise method. In addition, variables were candidates to enter the model whether they were shown to correlate to BE reversibility in literature or demonstrated to correlate to BE reversibility at univariate analysis in the present study, choosing a minimum of 4 to 5 events per variable [12]. The level of significance of the p-value of regression coefficients to enter was set to 0.05 and to exit the model at 0.10. Other variables associated with BE reversibility were thus tested step by step following the

same procedure to enter or exit the model. The process was stopped when all variables, enabled to both enter and stay into the model, were included. Conservative assessment of multicollinearity was performed to remove redundant information from the best model. Multicollinearity was detected whether the pair-wise coefficient of correlation between two variables was significantly superior to 0.6 and/or their variance inflation factor inferior to 5 [13]. Variables significantly cross-correlated were not entered into the same models to determine the best model predictive of exacerbations.

Table E1. CT characteristics

**Machine Brand**	**Kernel**	**kV**	**Slice thickness (mm)**
Somatom Sensation 16	B30f (n=11)	100	1
Somatom Definition 64	B30f (n=14)	(100-120)	1
Somatom Force	Br40d (n=11)	(100-110)	1
GE Revolution	STD (n=13)	120	1
Optima CT540	STD (n=3)	120	1

Kv : kilovolt, mm: millimeters

Table E2. Regional analysis of bronchial abnormalities and emphysema in patients with alpha1 antitrypsin deficiency.

	Upper lung		Lower lung		p-value
	Median	IQR	Median	IQR	
**Normalized Volumes**					
**Bronchiectasis**	8.4	[3.5; 22.8]	9.6	[4.2; 26]	<0.001
**Bronchial thickening**	5.3	[2.3; 13.7]	6.5	[3; 18]	<0.001
**Bronchial mucus**	0.1	[0; 0.8]	0.1	[0; 0.9]	0.01
**Bronchiolar mucus**	0	[0; 0.2]	0.1	[0; 0.2]	0.01
**Collapse/Consolidation**	1.5	[0.6; 8]	1.6	[0.6; 8.1]	<0.001
**Emphysema (LAV%))**	21.4	[12; 31]	22.5	[13; 36]	<0.001

Data correspond to median with IQR [interquartile range]. Comparison of paired medians were done with Wilcoxon rank test

The upper lung corresponds to the right and left upper lobes and middle lobe. The lower lung corresponds to the right and left lower lobes.

IQR: interquartile range, LAV: low attenuation value

Supplemental references

1. Dournes G, Hall CS, Willmering MM, et al (2022) Artificial intelligence in computed tomography for quantifying lung changes in the era of CFTR modulators. Eur Respir J 59:2100844. https://doi.org/10.1183/13993003.00844-2021
2. Bouzid AIH, De Senneville BD, Baldacci F, et al (2024) CT Evaluation of 2D and 3D Holistic Deep Learning Methods for the Volumetric Segmentation of Airway Lesions. In: 2024 IEEE International Symposium on Biomedical Imaging (ISBI). IEEE, Athens, Greece, pp 1–5
3. Hadj Bouzid AI, Bui S, Benlala I, et al (2024) Artificial intelligence-driven volumetric CT outcome score in cystic fibrosis: longitudinal and multicenter validation with/without modulators treatment. Eur Radiol. https://doi.org/10.1007/s00330-024-11019-5
4. Gupta S, Hartley R, Khan UT, et al (2014) Quantitative computed tomography-derived clusters: redefining airway remodeling in asthmatic patients. J Allergy Clin Immunol 133:729-738.e18. https://doi.org/10.1016/j.jaci.2013.09.039
5. Hadj Bouzid AI, de Senneville BD, Baldacci F, et al (2024) CT evaluation of 2D and 3D holistic deep learning methods for the volumetric segmentation of airway lesions
6. van Straten M, Brody AS, Ernst C, et al (2020) Guidance for computed tomography (CT) imaging of the lungs for patients with cystic fibrosis (CF) in research studies. J Cyst Fibros 19:176–183. https://doi.org/10.1016/j.jcf.2019.09.001

7. Quan K, Tanno R, Shipley RJ, et al (2019) Reproducibility of an airway tapering measurement in computed tomography with application to bronchiectasis. J Med Imaging (Bellingham) 6:034003. https://doi.org/10.1117/1.JMI.6.3.034003

8. Weikert T, Friebe L, Wilder-Smith A, et al (2022) Automated quantification of airway wall thickness on chest CT using retina U-Nets - Performance evaluation and application to a large cohort of chest CTs of COPD patients. Eur J Radiol 155:110460. https://doi.org/10.1016/j.ejrad.2022.110460

9. Mackin D, Ger R, Gay S, et al (2019) Matching and Homogenizing Convolution Kernels for Quantitative Studies in Computed Tomography. Invest Radiol 54:288–295. https://doi.org/10.1097/RLI.0000000000000540

10. Solomon JB, Christianson O, Samei E (2012) Quantitative comparison of noise texture across CT scanners from different manufacturers. Med Phys 39:6048–6055. https://doi.org/10.1118/1.4752209

11. Peduzzi P, Concato J, Kemper E, et al (1996) A simulation study of the number of events per variable in logistic regression analysis. J Clin Epidemiol 49:1373–1379. https://doi.org/10.1016/s0895-4356(96)00236-3

12. Vittinghoff E, McCulloch CE (2007) Relaxing the rule of ten events per variable in logistic and Cox regression. Am J Epidemiol 165:710–718. https://doi.org/10.1093/aje/kwk052

13. Grewal R, Cote JA, Baumgartner H (2004) Multicollinearity and Measurement Error in Structural Equation Models: Implications for Theory Testing. Marketing Science 23:519–529. https://doi.org/10.1287/mksc.1040.0070
